# The “glove” technique: a modified method for femoral fixation of antibiotic-loaded hip spacers

**DOI:** 10.3109/17453670903039551

**Published:** 2009-06-01

**Authors:** Konstantinos Anagnostakos, Daniel Köhler, Eduard Schmitt, Jens Kelm

**Affiliations:** Klinik für Orthopädie und Orthopädische Chirurgie, Universitätskliniken des SaarlandesHomburg/SaarGermany

## Introduction

In the past few years hip spacers have become popular in the treatment of infected total hip arthroplasties (THAs), with reported infection eradication rates of > 90% (Anagnostakos et al. 2006). Two methods have been described for femoral fixation of a hip spacer. The spacer stem can be inserted into the femur either by a “press-fit” method, or by cementing onto the proximal femur (Anagnostakos et al. 2006). It is unclear which of these two methods provides superior fixation and thus acts better in the prevention of a spacer dislocation. In this report, we introduce a new technique for femoral fixation of an antibiotic-impregnated hip spacer.

### Surgical technique

**Figure 1.**
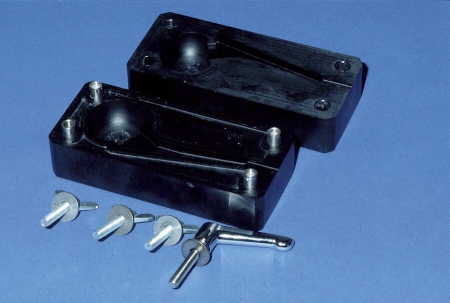
Mold, consisting of polyoxymethylene (POM), for standardized production of hip spacers. Each spacer has a head diameter of 50 mm, a stem length of 10 cm, and a total surface area of 13,300 mm^2^.

**Figure 2.**
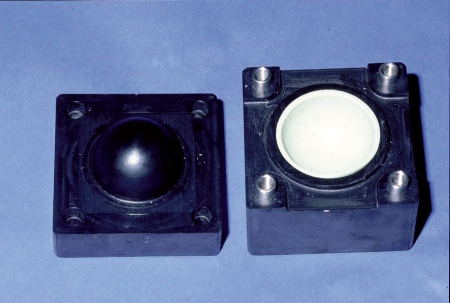
Mold for production of the acetabular component. The component has an inside/outside diameter of 53/56 mm and a total surface area of 4,410 mm^2^.

In our department, we produce hip spacers by means of a two-parted mold (Figure 1). The mold consists of polyoxymethylene (POM). A special mold is also available for acetabular defects (Figure 2). For clinical use, Refobacin-Palacos (0.5 g gentamicin/40 g cement) as bone cement has been shown to have elution characteristics that are better than those of other bone cements (Anagnostakos et al. 2006). For the spacer prosthesis and the acetabular component, production of 80 g and 40 g of polymethylmethacrylate (PMMA), respectively, is required. Depending on the identity of the causative pathogen and its sensitivity profile, we sometimes load the bone cement with a second antibiotic. In cases of an unidentified bacterium preoperatively or if the infection was revealed during an operation for presumed aseptic conditions, we routinelly use the combination of 1 g gentamicin/4 g vancomycin/80 g PMMA. Each spacer has a head diameter of 50 mm, a stem length of 10 cm, and a total surface area of 13,300 mm^2^. The acetabular component has an inside/outside diameter of 53/56 mm and a total surface area of 4,410 mm^2^. When there is to be a combination of antibiotics, the second antibiotic is added manually to the Refobacin-Palacos powder. After thorough mixing, the cement’s liquid monomer is added. After attaining a doughy state, the cement is then poured into the two halves of the spacer mold. The halves are then clamped together. After 15 min, they are opened again and the molded spacer is removed.

**Figure 3.**
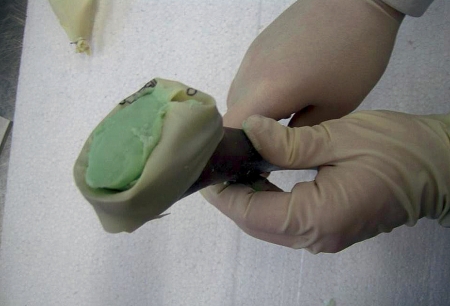
The antibiotic-loaded bone cement, consisting of the same mixture as the spacer, is introduced into the glove.

**Figure 4.**
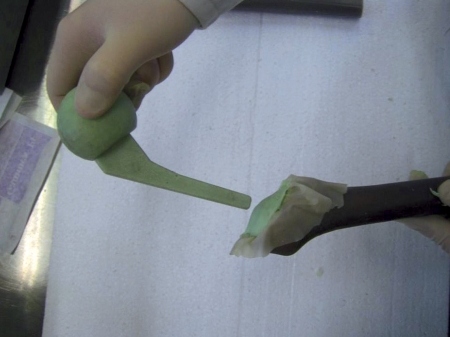
Insertion of the hip spacer into the cement-filled glove.

For femoral fixation of the spacer stem, we have developed the “glove” technique. A sterile glove is placed in the proximal femur, and the fingers are bound together with a vicryl suture. After preparing the same mixture as used for the spacer (40 g), the doughy bone cement is now introduced into the glove (Figure 3). Afterwards, the intraoperatively prepared spacer is inserted into the cement-filled glove (Figure 4).

**Figure 5.**
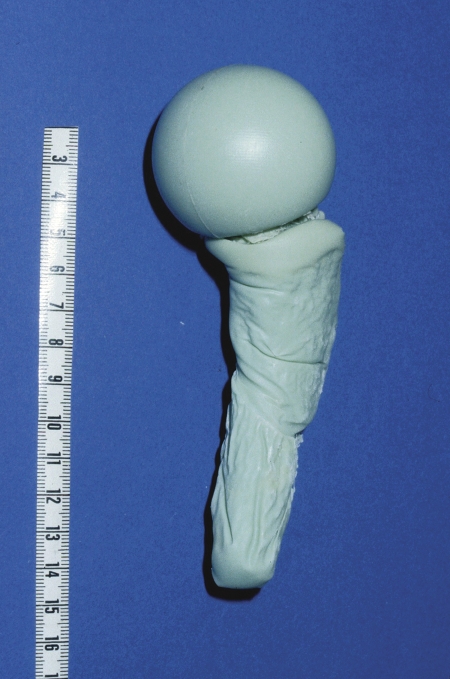
After removal of the glove, the cement mantle around the stem of the spacer shows an almost exact anatomical copy of the proximal part of the femur.

The entire construction is removed after a minimum of 2 min, yielding a spacer that is a nearly exact anatomical copy of the proximal femoral part (Figure 5). There is no risk that the spacer-glove complex will get stuck in the femur, as long as it is removed after 2 min—before the heat of polymerization has started.

**Figure 6.**
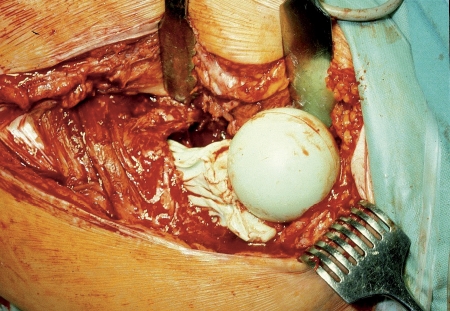
Reinsertion of the spacer into the femur.

**Figure 7.**
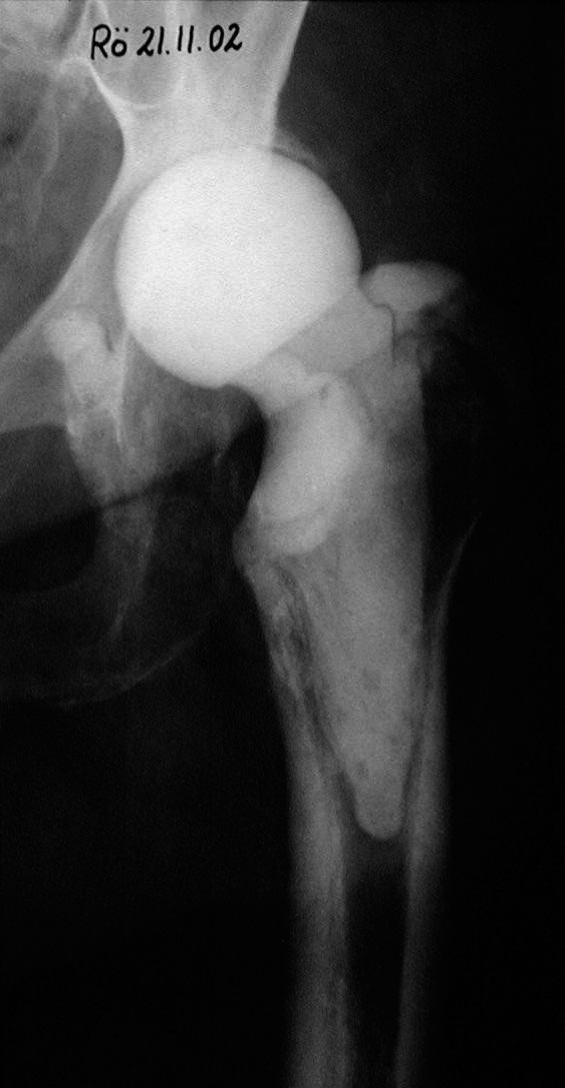
Articulating hip spacer fixed according to the “glove technique” in situ.

The glove is removed from the cement mantle around the spacer’s stem. Following the reinsertion of the stem (Figure 6), the remaining doughy cement is used for punctual fixation onto the femoral resection surface. After the cement has hardened, the spacer is reduced (Figure 7). For implantation of an acetabular spacer, this is normally cemented. We do not consider that it is necessary to use the glove technique also for the acetabular component because if a dislocation occurs, this happens on the femoral side. To our knowledge, there have been no reports about dislocation of an acetabular spacer and we have not seen any such case in our department.

## Discussion

The major factors that may influence the risk of dislocation of a hip spacer include the spacer’s geometry and its cervicodiaphyseal angle, the means of production (manually shaped vs. standard fit), the method of fixation to the proximal femur, and residual bone deficits of the acetabulum—to which the shape or size of the spacer cannot adapt. Despite the wide use of hip spacers and an estimated dislocation rate of 10–20%, there is no consensus on the ideal femoral fixation method.

There is controversial data in the literature regarding hip spacer dislocations. Leunig et al. (1998) reported dislocations of the hip in 5 of 12 patients after implantation of a hand-made spacer. The authors showed that with regard to the geometry a relatively small spacer femoral neck/head ratio should be aimed for (< 0.73), and deep insertion of the spacer into the femur is recommended (> 57 mm). Magnan et al. (2001) and Duncan et al. (1993) described 1/10 and 3/13 dislocations, respectively, after implantation of a standardized spacer. On the contrary, Ries and Jergesen (1999), Koo et al. (2001), Shin et al. (2002), and Takahira et al. (2003) did not observe any spacer dislocation in their respective series.

Despite the fact that there have been numerous reports about dislocations after hip spacer implantation, the precise method of fixation is often not well and accurately described in the literature. Although a proximal cementation of the spacer to the femur might preserve leg length and prevent any rotation compared to the “press-fit” method, there have been no studies showing which one of the two methods is superior. However, one possible disadvantage of normal cementation of a hip spacer may be that the cement can leach through the pre-drilled femoral canal so that difficulties can occur at the spacer explantation later, due to the cement removal. The “glove” technique allows spacer explantation in one piece without cement debris, thus reducing both mechanical complications and operating time. Over the past 7 years, we have observed that the “glove” technique gives a lower dislocation rate than the “press-fit” method, and that it allows a shorter reimplantation time compared with the “normal” cementation because no cement debris has to be removed from the femoral canal (unpublished data). Based on these observations, this technique has become a standard procedure in our department. Moreover, another advantage is that this technique can also be used for other, commercially available hip spacers such as the Spacer G or those made by using the Biomet silicon molds.
